# Turning over New Leaves

**Published:** 1890-02-22

**Authors:** 


### TURNING OVER NEW LEAVES.

### Two Health Resorts.*

HERE are two nicely got-up books, each putting forward the advantages of a health resort; one resort being in the far north, the other in the extreme south of Great Britain. Strathpeffer Spa being far less well-known than Bournemouth, we will first consider the concise guide to that place which lies before us. The "strath" of Peffer is in Cromarty, in one of those small pieces of that ridiculous Scotch county, which has struggled into the heart of Ross-shire. It is close under the shade of the mighty Ben Nevis, and the valley opens out to Dingwall, to let the fresh sea breezes come in. It is a beautiful country, rugged, wild, and quaint, and the invalid, tired of the endless promenades and sea fronts of our southern towns, can find quite a new existence in this out-of-the-way northern village. The climate is particularly exhilarating, and there are few cold winds, and very little mist or fog. In the summer the long days—in fact endless days, for the short nights are never quite dark there—are a great inducement to outdoor life, and though Strathpeffer in winter is of course colder than Bournemouth, it has its spells of warm sunshine brightening up the whole valley. The sulphur waters, massage baths, and chalybeate spring, are specially beneficial in certain cases of gout, rheumatism, and eczema. The second part of Dr. Fox's book is devoted to a pleasant description of the excursions in the neighbourhood, and to miscellaneous information which must be invaluable to the visitor to the Spa. The praises of Bournemouth have been frequently sung, but Mr. Kinsey Morgan has managed to gather together a great deal of fresh information of value to invalids, the chapter on Balneology being specially interesting. It is certainly a good thing that our physicians are beginning to point out the advantages of English health resorts, instead of sending their patients long journeys in search of that which they can obtain as well at home.

### Dad's Dorothy.*

This is a pretty tale for children, a book suitable for the children's ward, and suitable also for the schoolroom or the nursery. Perhaps the author is not quite correct in the earlier chapters in her spelling of Scotch words, and, like most writers, she is under the mistaken notion that the moon in any phase can be made to rise or set at any time. But these are mere details, and the story as a whole is as fresh and wholesome as anyone could wish; it is religious without being mawkish, and is exciting without being extravagant. The characters are very well drawn, especially that of the little fishermaiden. It is delightful to read about children who are natural, with good promptings in the naughty ones, and faults even in the hero and heroine. Some of the sayings scattered through the book show that the author is something more than a mere spinner of stories. For instance, the reply of the old butler to his mistress, who tells him boys will be boys—"Yes, my lady, so they will—and they couldn't be anythink wus."

* Dad's Dorothy, by M. B. Manwell. (Religious Tract Society.) Price 1s

